# Antimicrobial Stewardship in Vancomycin-Resistant *Enterococcus faecalis*: Clinical Study Evaluating Antimicrobial Susceptibility Patterns and Antibacterial Regimens

**DOI:** 10.3390/antibiotics15070672

**Published:** 2026-07-09

**Authors:** Samantha A. Tolbert, Mollie V. Day, Aline Arif, Preston H. Tolbert, Destyn Dicharry, Alexandre E. Malek

**Affiliations:** 1 Ochsner LSU Health Shreveport—Academic Medical Center, Shreveport, LA 71103, USA; 2 Division of Infectious Diseases, Department of Medicine, School of Medicine, LSU Health Shreveport, Shreveport, LA 71103, USA

**Keywords:** vancomycin-resistant enterococci, *Enterococcus faecalis*, antimicrobial stewardship, ampicillin susceptibility

## Abstract

**Background:** Vancomycin-resistant enterococci (VRE) *Enterococcus faecalis* (*E. faecalis)* remains largely ampicillin-susceptible, yet broad-spectrum agents are frequently used empirically (i.e., daptomycin and linezolid). Despite its frequent susceptibility to ampicillin, VRE *E. faecalis* is often managed with broad-spectrum antimicrobials, raising concerns about overtreatment and antimicrobial stewardship. Resistance mechanisms vary, with vancomycin resistance mediated by *vanA*/*vanB* operons and beta-lactams by altered penicillin binding proteins. These distinct mechanisms help explain why VRE *E. faecalis* isolates may remain susceptible to ampicillin, supporting its potential use in clinical isolates. This study aims to describe the antibacterial susceptibility results of *Enterococcus faecalis* VRE and assess antibiotics use in clinical settings in a tertiary medical center. **Methods:** This was a single-center, retrospective, descriptive study of hospitalized and outpatient adults with *Enterococcus faecalis* VRE isolates identified between 1 June 2018 and 15 March 2025. We excluded individuals with concomitant non-*Enterococcus faecalis* VRE infections and those aged <18 years. Vancomycin resistance was defined by a minimum inhibitory concentration of >32 mcg/mL per the 35th edition of the Clinical and Laboratory Standards Institution breakpoints. **Results:** A total of 337 patients were screened; 114 met inclusion criteria. The cohort was 54% female, 50% Black, and 48% White, with 54% having hypertension, 36% diabetes, and 19% a history of multidrug-resistant organism carriage. The most common source of infection was urinary (67%), followed by skin and soft tissue infections and bone/joint infections (11% each). All isolates were vancomycin-resistant; 82% were susceptible to ampicillin, 84% to nitrofurantoin, 61% to daptomycin, and 70% to linezolid. Antimicrobial regimens varied, with daptomycin being the most used agent (19%). No patients in the aminopenicillin group experienced 30-day mortality, whereas two patients (3%) in the non-aminopenicillin group died within 30 days. Acute kidney injury occurred in four patients (29%) in the aminopenicillin group compared with four patients (7%) in the non-aminopenicillin group, representing a significantly higher incidence in the aminopenicillin group (*p* = 0.037). However, myalgia, elevated creatine phosphokinase, and thrombocytopenia were more common in the non-aminopenicillin group. **Conclusions:** Despite higher susceptibility to ampicillin and nitrofurantoin, daptomycin was the most used agent for VRE *E. faecalis* infections. These findings highlight a need for improved antimicrobial stewardship and further clinical studies to guide optimal therapy.

## 1. Introduction

Infections caused by vancomycin-resistant enterococci (VRE) can range from localized infections, such as urinary tract infections, to more invasive diseases, notably intra-abdominal infections, bloodstream infections, and infective endocarditis [1]. Patients from whom VRE species are isolated often have a history of prolonged hospitalizations and prior use of multiple courses of antibiotics [1]. This organism is often intrinsically resistant to many antimicrobials (i.e., cephalosporins, antistaphylococcal penicillins, and sulfamethoxazole-trimethoprim) and thus is typically treated with aminopenicillins or vancomycin [2]. The incidence of VRE is continuously increasing in the United States and is mainly driven by the emergence of *Enterococcus faecium* (*E. faecium*), often resistant to both vancomycin and ampicillin [2,3]. In contrast to VRE *E. faecium*, VRE *Enterococcus faecalis* (*E. faecalis*) isolates remain primarily ampicillin-susceptible [1,4]. However, in clinical practice when VRE *E. faecalis* is isolated, practitioners often default to the administration of broad-spectrum antibiotic therapy such as daptomycin or linezolid [5].

Generally, there are five types of vancomycin resistance, including *vanA*, *vanB*, *vanC*, *vanD*, *vanE*. Most are acquired mechanisms, except *vanC* which is intrinsically encoded chromosomally. These genes block the inhibition of cell wall synthesis and act at the peptidoglycan terminus of the bacterial cell wall, where D-Ala-D-Ala is typically targeted. Species intrinsically carrying *vanC* include *E. gallinarum* and *E. casseliflavus*/*E. flavescens*. *E. faecalis* becomes vancomycin-resistant by acquiring *vanA*, *vanB*, or *vanE*, which block the inhibition of cell-wall synthesis while remaining susceptible to ampicillin [2]. *E. faecalis* can produce penicillinase, causing non-susceptibility to antistaphylococcal penicillins but remaining susceptible to ampicillin, amoxicillin, and piperacillin in a standard inoculum infection [4,6]. In high inoculum, preclinical studies suggested that the addition of a beta-lactamase inhibitor may counteract these beta-lactamases [1,4]. The resistant mechanism for ampicillin in *E. faecium* is different as this organism expresses PBP5 mutation [1,4]. These diverse resistance mechanisms highlight why a narrow-spectrum agent like ampicillin can remain effective against VRE *E. faecalis*. The SENTRY data showed that all 254 VRE *E. faecalis* isolates were ampicillin-susceptible, although clinical evidence supporting its use is limited to urinary tract infection [7,8,9,10]. The primary objective of this study was to compare 30-day mortality in patients with VRE *E. faecalis* who received aminopenicillin-based therapy versus those who received non-aminopenicillin therapy. The secondary outcomes included rates of recurrent infection within 30 days and adverse drug events such as acute kidney injury, myalgia, elevated creatine phosphokinase, and thrombocytopenia during the duration of treatment.

## 2. Results

### 2.1. Population Characteristics and Baseline Demographics

A total of 337 patients were screened, and 114 met the inclusion criteria. The median age of the cohort was 63 years (range, 18–99); 62 patients (54%) were female, 57 (50%) were Black, and 55 (48%) were White. Common comorbidities included hypertension in 62 patients (54%), diabetes mellitus in 41 (36%), hyperlipidemia in 25 (22%), chronic kidney disease in 19 (17%), and human immunodeficiency virus in 2 (2%) (Table 1). Prior hospitalizations within the preceding three months were reported in 51 patients (45%). Multidrug-resistant organism carriage within the past year of hospitalization was as follows: extended spectrum beta lactamases Enterobacterales 9 (8%), methicillin-resistant *Staphylococcus aureus* 6 (5%), vancomycin-resistant *Enterococcus faecium* and/or *Enterococcus faecalis* 3 (7%), and *Stenotrophomonas maltophilia* 2 (2%).

### 2.2. Microbiology and Infectious Source

All isolates were resistant to vancomycin; 93 (82%) remained susceptible to ampicillin, 13 (76%) to penicillin, 63 (84%) to nitrofurantoin, 67 (61%) to daptomycin, and 80 (70%) to linezolid (Table 2). All percentages represent the total tested, as some patients did not have selected antimicrobial available during susceptibility testing. The most common co-pathogens were Enterobacterales species in 55 patients (48%), followed by *Pseudomonas aeruginosa* in 13 (11%), *Staphylococcus aureus* in 7 (6%), *Acinetobacter baumannii* in 6 (5%), *Streptococcus* species in 6 (5%), and *Candida* species in 6 (5%). The *Enterococcus* species alone was isolated in 38 patients (34%), and 37 (32%) were determined to represent colonization. Total isolates were obtained from the following sources: 76 (67%) were from the urinary tract, 13 (11%) skin and soft tissue, 12 (11%) bone/joint, 9 (8%) blood, and 4 (4%) intraabdominal.

### 2.3. Clinical Features and Outcomes

All VRE *E. faecalis* isolates that were deemed to represent colonization or contamination were excluded from the primary and secondary outcome analyses, resulting in 77 patients included in the final analysis for primary and secondary outcomes. The most commonly used antimicrobial was daptomycin in 22 patients (19%) followed by aminopenicillins in 20 patients (17%) (Table 3). Fourteen patients received an aminopenicillin for the entire treatment course, while 63 patients in the non-aminopenicillin group received a broad range of therapies. Clinical sources of infection differed between the aminopenicillin and non-aminopenicillin groups, respectively, as follows: urinary sources (64% vs. 56%), skin and soft tissue infections (7% vs. 17%), bone/joint infections (0% vs. 14%), bloodstream infections (21% vs. 8%), and intra-abdominal infections (7% vs. 3%) (Table 4). Additionally, one *Enterococcus faecalis* isolate was recovered from an oral cavity source in the non-aminopenicillin group. The patient received antimicrobial therapy because of an extensive surgical procedure in the setting of concurrent clinical signs and symptoms of infection. Readmission within 30 days rates were similar between the two groups: 3 patients (21%) in the aminopenicillin group and 15 patients (21%) in the non-aminopenicillin group. Thirty-day mortality was zero in the aminopenicillin group and occurred in two patients (3%) in the non-aminopenicillin group. Acute kidney injury occurred more frequently in the aminopenicillin group (4 patients, 29%; *p =* 0.037) compared to the non-aminopenicillin group (4 patients, 7%). However, myalgia, elevated creatine phosphokinase, and thrombocytopenia were more common in the non-aminopenicillin group (Table 5).

## 3. Discussion

Despite the higher susceptibility of VRE *E. faecalis* to ampicillin and penicillin, daptomycin remained the most frequently used agent at our institution. These findings highlight ongoing opportunities for antimicrobial stewardship and underscore the need for additional clinical studies to better define optimal empiric and targeted treatment strategies. To the best of our knowledge, this is the first clinical study to evaluate aminopenicillin use for VRE *E. faecalis* infections across multiple infection sites, although the majority of isolates in our cohort originated from urinary tract sources. When isolates demonstrate aminopenicillin susceptibility and patient-specific factors such as adequate site penetration, tolerability, and insurance coverage are favorable, aminopenicillins may represent a reasonable narrower-spectrum alternative to broader agents in selected clinical scenarios. This concept may be particularly relevant in empiric treatment considerations for VRE *E. faecalis* infections. The SENTRY database reported 100% ampicillin susceptibility among VRE *E. faecalis* isolates, whereas in our cohort, 93 isolates (82%) remained susceptible to ampicillin and 13 (76%) to penicillin, with comparatively lower susceptibility to daptomycin and linezolid (61% and 70%, respectively) [7].

When evaluating infection-specific guidelines, most recommend broad-spectrum agents such as daptomycin or linezolid for VRE infections without differentiating between VRE *Enterococcus faecalis* and VRE *Enterococcus faecium* or consider retained aminopenicillin susceptibility in select isolates. The clinical literature in this area remains limited, highlighting an important gap regarding empiric and targeted treatment strategies for ampicillin-susceptible VRE *E. faecalis* infections. Most published clinical data involving aminopenicillins for VRE focus on urinary tract infections; however, guidelines do not provide specific recommendations for VRE *E. faecalis* ampicillin susceptible isolates [11,12,13,14]. Heintz and colleagues supported the use of aminopenicillins for VRE urinary tract infections despite elevated aminopenicillin MICs, likely due to renal tubular secretion producing urinary concentrations substantially higher than serum level [8]. Similarly, Shah and colleagues reported an 88.1% clinical cure rate in VRE cystitis caused by ampicillin-nonsusceptible isolates [10]. Cole and colleagues also demonstrated comparable outcomes between aminopenicillins and non-beta-lactam therapy for VRE infections regardless of aminopenicillin susceptibility, with clinical cure achieved in 83.9% versus 73.3% of patients, respectively [15]. In our cohort, urinary tract infections represented the most common infectious source, accounting for 64% of isolates in the aminopenicillin group and 56% in the non-aminopenicillin group, with a minority concomitantly infected with bacteremia. According to the guideline recommendations for non-urinary VRE infections, these similarly favor broader agents without specifically addressing aminopenicillin-susceptible VRE *E. faecalis* isolates. The 2014 skin and soft tissue infection guidelines recommend daptomycin, linezolid, or colistin for VRE infections, although aminopenicillin-containing regimens such as ampicillin-sulbactam and piperacillin-tazobactam are discussed for necrotizing infections and amoxicillin-clavulanate is recommended for bite wounds, supporting adequate aminopenicillin tissue penetration [16]. Likewise, the 2015 vertebral osteomyelitis guidelines recommend daptomycin or linezolid for VRE while noting penicillin as an option for susceptible *Enterococcus* species but do not specifically address ampicillin-susceptible VRE *E. faecalis* [17]. Although there was no infective endocarditis cases identified in our cohort, this infection source remains clinically important because current guideline interpretations leave room for consideration of ampicillin-based therapy in select VRE *E. faecalis* isolates. The American guidelines recommend ampicillin or penicillin plus gentamicin, or dual beta-lactam therapy with ampicillin plus ceftriaxone, for enterococcal endocarditis caused by penicillin-susceptible isolates but do not clarify whether these recommendations apply to vancomycin-resistant isolates that remain aminopenicillin susceptible [18]. In contrast, European guidelines recommend combination therapy for VRE endocarditis using daptomycin with agents such as ampicillin, ertapenem, ceftaroline, or fosfomycin, although they similarly do not distinguish between *Enterococcus* species [19]. Supporting this concept, Funk and colleagues demonstrated enhanced bacterial killing with ampicillin-ceftobiprole combination therapy against borderline penicillin-resistant, ampicillin-susceptible, vancomycin-resistant *E. faecalis* isolates in vitro, suggesting that aminopenicillins may remain viable in combination regimens for select invasive infections [20]. Additional guideline gaps exist for catheter-related bloodstream and intra-abdominal infections. Catheter-related infection guidelines recommend ampicillin for ampicillin-susceptible *Enterococcus* species and daptomycin or linezolid for VRE, without differentiating between *Enterococcus* species or retained aminopenicillin susceptibility [21]. Similarly, intra-abdominal infection guidelines distinguish *E. faecalis* from *E. faecium* but do not specifically address ampicillin-susceptible VRE *E. faecalis* isolates [22]. Interestingly, meningitis guidelines remain among the few to differentiate between ampicillin-susceptible and ampicillin-resistant VRE isolates, recommending ampicillin plus gentamicin for ampicillin-susceptible VRE infections, likely due to the enhanced antibiotic penetration of the blood–brain barrier compared to other agents such as daptomycin [23].

Daptomycin-susceptible dose-dependent (SDD) breakpoints (MIC ≤ 4 mg/L) are established for *Enterococcus faecium*; however, no SDD breakpoints currently exist for non-*E. faecium Enterococcus* species, including *E. faecalis* [24]. Instead, CLSI categorizes intermediate breakpoints as those with an MIC of 4 mg/L. In our cohort, 16% of isolates demonstrated daptomycin MICs interpreted as intermediate. Although higher daptomycin doses may be effective against these isolates, CLSI dosing recommendations for SDD interpretations (8–12 mg/kg/day) are limited to *E. faecium*. Drake and colleagues suggested that high-dose daptomycin (≥8 mg/kg/day) may be a reasonable treatment option for *E. faecalis* isolates categorized as intermediate [25]. Applying this interpretation to our cohort would increase the proportion of isolates potentially treatable with daptomycin to 81%, which mirrors the proportion of isolates that were susceptible to ampicillin (82%). These findings suggest that both daptomycin and aminopenicillins may represent reasonable empiric treatment options for *E. faecalis* VRE infections pending susceptibility results.

## 4. Limitations

Our study has several limitations. As a retrospective study, confounders could not be fully controlled. Many patients had polymicrobial infections requiring broader-spectrum antimicrobial therapy, and several patients were lost to follow-up or lacked documented treatment plans. In addition, not all isolates underwent susceptibility testing against all antimicrobials because the antimicrobials included varied based on the MicroScan panel used at the time of testing. We strictly defined the aminopenicillin group and excluded patients who were initially treated with daptomycin or linezolid before de-escalation. Only 77 patients had assessable clinical outcomes, limiting the sample size. We observed higher 30 day all-cause mortality in the non-aminopenicillin group (two patients, 3%) compared with no deaths in the aminopenicillin group. Adverse effects were similar between groups, except for a higher incidence of acute kidney injury in the aminopenicillin group, which may be attributable to the small sample size and two patients concomitantly receiving nephrotoxic agents, including colistin and gentamicin. Finally, we did not perform a subgroup analysis comparing monomicrobial and polymicrobial *E. faecalis* infections. Consequently, we were unable to determine the extent to which concomitant pathogens influenced empiric antimicrobial selection or clinical outcomes. Future studies evaluating monomicrobial *E. faecalis* infections separately may better define the role of aminopenicillins in these infections.

## 5. Methods

### 5.1. Design

Single center, retrospective, descriptive chart review study between 1 June 2018, and 15 March 2025.

### 5.2. Population

All adult patients aged ≥18 years with VRE *E. faecalis* positive culture isolated from any type of specimens who were hospitalized or seen in the ambulatory clinic at Ochsner LSU Shreveport—Academic Medical Center. Patients with concomitant non-*E. faecalis Enterococcus* species or those belonging to sensitive populations (i.e., incarcerated, or pregnant individuals) were excluded. All variables were manually abstracted from the patient chart using the EPIC electronic medical record. Patients were included based on their first VRE *E. faecalis* isolation only.

### 5.3. Definitions

Vancomycin resistance was defined as a minimum inhibitory concentration (MIC) ≥ 32 mcg/mL. Susceptibility thresholds were defined as ≤8 mcg/mL for ampicillin and penicillin, ≤32 mcg/mL for nitrofurantoin, and ≤2 mcg/mL for daptomycin and linezolid, according to the 35th edition of the Clinical and Laboratory Standards Institute breakpoints [11]. Aminopenicillin therapy included ampicillin, piperacillin–tazobactam, amoxicillin, ampicillin–sulbactam, and amoxicillin–clavulanate. Non-aminopenicillin therapy consisted of daptomycin, linezolid, nitrofurantoin, fluoroquinolones, ceftaroline, tigecycline, and/or meropenem. Patients in the aminopenicillin group were treated with these agents during the entire treatment course. Susceptibility was performed using the MicroScan Walk Away method, which may or may not have penicillin or daptomycin results, depending on the cards available during the time of testing. Nitrofurantoin susceptibilities were only reported from urine cultures. All isolates deemed to be contaminants were excluded from the primary and secondary outcome analyses; however, susceptibility data were still evaluated.

### 5.4. Statistical Analysis

Statistical analysis was performed with Fisher’s exact test to serially compare the incidence of 30-day mortality and the incidence of secondary outcomes between intervention and control groups. All statistical analyses were performed using Python 3.12.12 through Google Colaboratory [26,27].

## 6. Conclusions

In this single-center retrospective study, 82% of VRE *E. faecalis* isolates retained aminopenicillin susceptibility, yet broad-spectrum therapies remained the predominant treatment strategy. These findings support further investigation into susceptibility-guided treatment approaches and reinforce the importance of antimicrobial stewardship in the management of VRE *E. faecalis*. Aminopenicillins may be considered in selected clinical scenarios when microbiologic susceptibility and patient-specific factors are favorable; however, larger prospective studies are needed before broader conclusions regarding efficacy across infection sources can be made.

## Figures and Tables

**Table 1:** Characteristics of the patients, infections, and co-pathogens.

Characteristics	Overall; *n* = 114
Age, median (range)	63 (18–99)
Female sex—no. (%)	62 (54)
Race and Ethnicity—no. (%)	
Black	57 (50)
White	55 (48)
Hispanic or Latino	2 (2)
Coexisting conditions—no. (%)	
Hypertension	62 (54)
Diabetes mellitus	41 (36)
Hyperlipidemia	25 (22)
Chronic kidney disease/end stage renal disease	19 (17)
Congestive heart failure	14 (12)
Cancer	12 (11)
HIV/AIDS *	2 (2)
Hemodialysis—no. (%)	21 (19)
Chronic vascular catheter—no. (%)	
Short Term	27 (24)
Long Term	71 (62)
Port-a-cath	12 (11)
Present surgical history—no. (%)	
Joint/bone hardware	10 (9)
Colostomy	9 (8)
Urostomy or PCN ^†^	9 (8)
Prior hospitalization within past three months	51 (45)
MDRO ^‡^ carriage within one year—no. (%)	
Extended spectrum β lactamases organisms	9 (8)
Methicillin resistant *Staphylococcus aureus*	6 (5)
Vancomycin-resistant Enterococci	3 (7)
*Stenotrophomonas maltophilia*	2 (2)
Carbapenem resistance *Acinetobacter* baumannii	1 (1)
Multi-drug-resistant *Pseudomonas aeruginosa*	1 (1)
Infectious source—no. (%)	
Urine	76 (67)
Skin and soft tissue	13 (11)
Osteoarticular	12 (11)
Blood	9 (8)
Intraabdominal	4 (4)
Respiratory	2 (2)
Cerebral spinal fluid	1 (1)
Mouth	1 (1)
Co-pathogens—no. (%)	
Enterobacterales ^§^	55 (48)
*Pseudomonas aeruginosa*	13 (11)
*Staphylococcus aureus* ^¶^	7 (6)
*Acinetobacter baumannii*	6 (5)
*Streptococcus* spp. ^^^	6 (5)
*Candida* spp ^‖^	6 (5)
*Stenotrophomonas maltophilia*	3 (3)
*Pseudomonas fluorescens*/*putida*	3 (3)
*Bacteroides fragilis*	1 (1)
Monomicrobial *Enterococcus faecalis*	39 (34)

* HIV/AIDS: Human immunodeficiency virus/acquired immunodeficiency syndrome, ^†^ PCN: percutaneous nephrostomy, ^‡^ MDRO: multidrug-resistant organisms, ^§^ Enterobacterales: *Escherichia coli* (*n* = 16), *Klebsiella pneumoniae* (*n* = 14), *Proteus mirabilis* (*n* = 9), *Morganella morganii* (*n* = 6), *Providencia stuartii* (*n* = 4), *Serratia marcescense* (*n* = 3), *Enterobacter cloacae* complex (*n* = 1), *Proteus vulgaris* (*n* = 1), *Providencia rettgeri* (*n* = 1), ^¶^ *Staphylococcus aureus*: Methicillin-resistant *Staphylococcus aureus* (*n* = 4), Methicillin susceptible *Staphylococcus aureus* (*n* = 3), ^‖^ *Candida* spp.: *Candida albicans* (*n* = 4), *Candida parapsilosis* (*n* = 1), *Candida tropicalis* (*n* = 1), ^^^ *Streptococcus* spp.: *Streptococcus* viridans group (*n* = 2), *Streptococcus pneumoniae* (*n* = 2), *Streptococcus pyogenes* (*n* = 1), Group C Streptococcus (*n* = 1).

**Table 2:** Vancomycin-resistant *Enterococcus faecalis* susceptibility pattern.

Antimicrobial and Minimum Inhibitory Concentration Breakpoints	Interpretation Results
Vancomycin (*n* = 114);—no. (%)	
Resistant (≥32 mcg/mL)	114 (100)
Penicillin (*n* = 17);—no. (%)	
Susceptible (≤8 mcg/mL)	13 (76)
Resistant (>16 mcg/mL)	4 (23)
Ampicillin (*n* = 114);—no. (%)	
Susceptible (≤8 mcg/mL)	93 (82)
Resistant (>16 mcg/mL)	21 (18)
Nitrofurantoin (*n* = 75);—no. (%)	
Susceptible (≤32 mcg/mL)	63 (84)
Intermediate (64 mcg/mL)	5 (7)
Resistant (>128 mcg/mL)	7 (9)
Tetracycline (*n* = 108);—no. (%)	
Susceptible (≤4 mcg/mL)	9 (8)
Resistant (>16 mcg/mL)	99 (92)
Daptomycin (*n* = 109);—no. (%)	
Susceptible (≤2 mcg/mL)	67(61)
Intermediate (4 mcg/mL)	22 (20)
Resistant (>8 mcg/mL)	20 (18)
Linezolid (*n* = 114);—no. (%)	
Susceptible (≤2 mcg/mL)	80 (70)
Intermediate (4 mcg/mL)	2 (2)
Resistant (>8 mcg/mL)	32 (28)
Gentamicin (*n* = 43);—no. (%)	
Susceptible (≤500 mcg/mL)	23 (53)
Resistant (>500 mcg/mL)	20 (46)
Levofloxacin (*n* = 22);—no. (%)	
Susceptible (≤2 mcg/mL)	4 (18)
Resistant (>8 mcg/mL)	18 (81)

Antimicrobial susceptibility testing was performed using the MicroScan Walk Away system. As antimicrobial agents included on the MicroScan panels varied by testing card, not all isolates underwent susceptibility testing for every agent. Therefore, susceptibility percentages were calculated using the number of isolates tested for each individual antimicrobial as the denominator.

**Table 3:** Antimicrobial regimens, patients without antibiotics, and antibiotic duration.

Antimicrobial Therapy	Overall *n* = 77
Antimicrobial—no. (%)	
Daptomycin	22 (19)
Aminopenicillins *	20 (17)
Carbapenem ^‖^	20 (17)
Nitrofurantoin	19 (17)
Non-fifth-generation or novel cephalosporin ^¶^	19 (17)
Vancomycin	12 (11)
Fluoroquinolone ^§^	10 (9)
Linezolid	8 (7)
Ceftaroline	3 (3)
Tigecycline	3 (3)
Other ^^^	10 (9)
No antibiotics—no. (%)	26 (22)
Antibiotic duration in days, median (range)	13 (1–132)

* Aminopenicillins: ampicillin, piperacillin-tazobactam, amoxicillin, ampicillin-sulbactam, amoxicillin-clavulanate, ^§^ Levofloxacin, ciprofloxacin, moxifloxacin, ^¶^ Non-fifth-generation or novel cephalosporin: cefepime, cephalexin, ceftriaxone, cefdinir, ^‖^ Carbapenem: meropenem, ertapenem, ^^^ Other: amikacin, gentamicin, ceftolozane-tazobactam, cefiderocol, colistin, metronidazole, sulfamethoxazole-trimethoprim, fosfomycin, micafungin, fluconazole.

**Table 4:** Clinical source of infection.

	Aminopenicillin Group ^a^	Non Aminopenicillin Group ^b^
	*n* = 14	*n* = 63
Urine	9 (64)	35 (56)
Skin and soft tissue	1 (7)	11 (17)
Bone/joint	0 (0)	9 (14)
Blood; Source	3 (21)	5 (8)
Catheter/Line	1 (33)	2 (40)
Urinary	1 (33)	2 (40)
Bone/Joint	0 (0)	1 (20)
Unknown	1 (33)	0 (1)
Intraabdominal	1 (7)	2 (3)
Oral Cavity	0 (0)	1 (2)

^a^ Aminopenicillin therapy included ampicillin, piperacillin–tazobactam, amoxicillin, ampicillin–sulbactam, and amoxicillin–clavulanate, ^b^ Non-aminopenicillin therapy consisted of daptomycin, linezolid, nitrofurantoin, fluoroquinolones (ciprofloxacin, levofloxacin), ceftaroline, tigecycline, and/or meropenem.

**Table 5:** Clinical outcomes and adverse drug events *n* = 77.

Variable—No. (%)	Aminopenicillin Group ^a^ *n* = 14	Non Aminopenicillin Group ^b^ *n* = 63	*p* Value
30 day mortality—no. (%)	0 (0)	2 (3)	1.000
Admitted within 30 days—no. (%)	3 (21)	15 (21)	1.000
Recurrent infection—no. (%)	1 (7)	4 (6)	1.000
AKI—no. (%)	4 (29)	5 (7)	0.037
Myalgia—no. (%)	0 (0)	2 (3)	1.000
Elevated CPK—no. (%)	0 (0)	1 (1)	1.000
Thrombocytopenia—no. (%)	0 (0)	5 (7)	0.585

Abbreviations: AKI: Acute kidney injury, CPK: Creatine Phosphokinase, ^a^ Aminopenicillin therapy included ampicillin, piperacillin–tazobactam, amoxicillin, ampicillin–sulbactam, and amoxicillin–clavulanate, ^b^ Non-aminopenicillin therapy consisted of daptomycin, linezolid, nitrofurantoin, fluoroquinolones (ciprofloxacin, levofloxacin), ceftaroline, tigecycline, and/or meropenem.

## Data Availability

The data presented in this study are available upon request from the corresponding author due to the agreement with the Institutional Review Board to share information relevant only publicly to the study, which is presented in the article.
